# Production of Long Chain Fatty Alcohols Found in Bumblebee Pheromones by *Yarrowia lipolytica*

**DOI:** 10.3389/fbioe.2020.593419

**Published:** 2021-01-08

**Authors:** Jaroslav Hambalko, Peter Gajdoš, Jean-Marc Nicaud, Rodrigo Ledesma-Amaro, Michal Tupec, Iva Pichová, Milan Čertík

**Affiliations:** ^1^Faculty of Chemical and Food Technology, Institute of Biotechnology, Slovak University of Technology, Bratislava, Slovakia; ^2^French National Research Institute for Agriculture (INRAE), Food and Environment, AgroParisTech, Micalis Institute, Université Paris-Saclay, Jouy-en-Josas, France; ^3^Department of Bioengineering and Imperial College Centre for Synthetic Biology, Faculty of Engineering, Imperial College London, London, United Kingdom; ^4^Institute of Organic Chemistry and Biochemistry of the Czech Academy of Sciences, Prague, Czechia

**Keywords:** pheromone, fatty alcohol, reductase, metabolic engineering, *Bombus*, *Yarrowia lipolytica*

## Abstract

Fatty alcohols (FA-OH) are aliphatic unbranched primary alcohols with a chain of four or more carbon atoms. Besides potential industrial applications, fatty alcohols have important biological functions as well. In nature, fatty alcohols are produced as a part of a mixture of pheromones in several insect species, such as moths, termites, bees, wasps, etc. In addition, FA-OHs have a potential for agricultural applications, for example, they may be used as a suitable substitute for commercial insecticides. The insecticides have several drawbacks associated with their preparation, and they exert a negative impact on the environment. Currently, pheromone components are prepared mainly through the catalytic hydrogenation of plant oils and petrochemicals, which is an unsustainable, ecologically unfriendly, and highly expensive process. The biotechnological production of the pheromone components using engineered microbial strains and through the expression of the enzymes participating in the biosynthesis of these components is a promising approach that ensures ecological sustenance as well. The present study was aimed at evaluating the production of FA-OHs in the oleaginous yeast, *Yarrowia lipolytica*, with different lengths of fatty-acyl chains by expressing the fatty acyl-CoA reductase (FAR) *Blap*FAR4 from *B. lapidarius*, producing C16:0-OH, C16:1Δ^9^-OH, and lower quantities of both C14:0-OH and C18:1Δ^9^-OH, and *Bluc*FAR1 from *B. lucorum*, producing FA-OHs with a chain length of 18–26 carbon atoms, in this yeast. Among the different novel *Y. lipolytica* strains used in the present study, the best results were obtained with JMY7086, which carried several lipid metabolism modifications and expressed the *BlucFAR1* gene under the control of a strong constitutive promoter *8UAS-pTEF*. JMY7086 produced only saturated fatty alcohols with chain lengths from 18 to 24 carbon atoms. The highest titer and accumulation achieved were 166.6 mg/L and 15.6 mg/g DCW of fatty alcohols, respectively. Unlike JMY7086, the *Blap*FAR4-expressing strain JMY7090 produced only 16 carbon atom-long FA-OHs with a titer of 14.6 mg/L.

## Introduction

Fatty alcohols (FA-OH) are aliphatic unbranched primary alcohols with varying chain lengths ranging from 4 to 28 carbon atoms and containing either saturated or unsaturated carbon bonds (McNaught and Wilkinson, 1997). The properties and the potential applications of fatty alcohol depend on their molecular structure. Generally, fatty alcohols are used as fuels, solvents, detergents, cosmetics, lubricants, and pharmaceuticals, or may serve as precursors for other compounds such as waxes or polymers (Rutter and Rao, 2016; Wang G. et al., 2016; Wang W. et al., 2016; Borodina et al., 2018a; Cordova et al., 2019). In 2019, the global demand for fatty alcohols was estimated to be over two million tons, with an annual growth rate of 4.3%. Traditionally, these molecules are produced through the catalytic hydrogenation of petrochemicals or plant oils, which currently relies on fossil fuels or unsustainable palm farming and has significant environmental consequences such as deforestation or contribution to global warming (Rutter and Rao, 2016; Shah et al., 2016; Cordova et al., 2019). Therefore, there is an urgent requirement for a further efficient and ecologically-friendly process.

Fatty alcohols and their derivatives also have important biological functions. Insects have evolved an efficient mate-finding system that is based on volatile sex pheromones. In most species, sex pheromones are released either as a single component (Jurenka, 2004; Groot et al., 2016; Tupec et al., 2017) or as a specific blend of molecules in specific ratios, most of which are fatty acid (FA) derivatives, usually alcohols, aldehydes, or acetates. The sex pheromone blend of the bumblebees mainly comprises saturated, mono-unsaturated, and poly-unsaturated fatty alcohols with a chain length of 16–18 carbon atoms along with terpenoid compounds (Ayasse and Jarau, 2014). Sex pheromones are synthesized *de novo* in specialized cells known as pheromone glands, which have evolved from epidermal cells (Žáček et al., 2013; Tittiger and Blomquist, 2017). The pheromone biosynthesis process involves several key enzymes. In addition, the saturated FAs such as stearic and palmitic must undergo processing mediated by chain-shortening enzymes, desaturases, reductases, acetyltransferases, and oxidases, among others (Tillman et al., 1999; Jurenka, 2004; Matoušková et al., 2008; Matsumoto, 2010; Buček et al., 2013; Koutroumpa and Jacquin-Joly, 2014; Tupec et al., 2017).

The first pheromone to be identified and purified was a fatty alcohol named bombykol (10*E*,12*Z*- hexadeca-10,12-dien-1-ol), which was isolated from the silkmoth *Bombyx mori* (Butenandt et al., 1961). Enzymes involved in bombykol biosynthesis were described later (Moto et al., 2003, 2004). Since then, pheromones have been identified in several thousands of insect species and are known for their potential as attractants or repellents in agriculture (Koutroumpa and Jacquin-Joly, 2014; El-Sayed, 2019). Pheromones represent the most suitable substitute for insecticides. Insecticides have been in use in agriculture for over 50 years. However, the environmental damage caused by the insecticides and the development of insecticide resistance among insects and pests are emerging as serious threats. The most significant problems associated with the use of insecticides include: (a) harmful effects on other organisms, including humans and the plants that rely on insects for pollination, (b) persistence of the insecticides in the biosphere, (c) worldwide spread, and (d) significant levels of pollution associated with the current methods of insecticide production (Hagström et al., 2013; Borodina et al., 2018b). In order to resolve some of these issues, synthetic pheromones were developed to control the pest insects in a species-specific manner and to maintain healthy agricultural practices (Hagström et al., 2013). For instance, tetradec-9-enyl acetate (C14:1Δ^9^-OAc) was reported to disrupt the mating efficiency of the fall armyworm when applied alone (Mitchell and McLaughlin, 1982). However, most agricultural applications of pheromones are limited by their high cost. Pheromones are expensive because their purity is paramount for eliciting a response in an insect, and the production of pheromones with such high levels of purity through chemical processes requires expensive and complicated methods while generating waste in huge quantities, which again requires disposal and increases costs (Borodina et al., 2018b).

The development of various genetic tools has allowed the characterization of heterologously produced fatty acyl-CoA reductases (FARs), which catalyze the reduction of fatty acyl-CoA precursors into the corresponding alcohols (Moto et al., 2003; Liénard et al., 2010; Antony et al., 2016; Ding et al., 2016; Tupec et al., 2019). The FAR genes are present in several species, including vertebrates, non-insect invertebrates, and fungi, with a particularly high number of FAR gene families reported in plant and insect genomes (Eirín-López et al., 2012; Buček et al., 2016; Tupec et al., 2019). Within the class Insecta, large quantities of long-chain alcohols have been identified in the pheromone mixtures of different bumblebees, including *Bombus lucorum*, *Bombus lapidarius*, and *Bombus terrestris*. Tupec et al. (2019) used heterologous expression in *S. cerevisiae* to demonstrate that bumblebees have evolved a specific FAR gene group that encodes reductases with unusual specificities and contributes to the biosynthesis of different fatty alcohols that form a part of bumble-specific pheromones.

The recent advancements in metabolic engineering and synthetic biology have enabled an environment-friendly production of FA-derived compounds, including FA-OHs and the biofuels from renewable feedstock using microbial biomass (Guo et al., 2016; Rutter and Rao, 2016). *Yarrowia lipolytica* is an oleaginous non-pathogenic yeast belonging to the Ascomycota phylum of kingdom Fungi (Abdel-Mawgoud et al., 2018), which could serve as a perfect cell factory for industrial applications (Groenewald et al., 2014). This yeast species is of great importance to researchers due to its high tolerance to a variety of organic substrates, higher salt levels in the environment, and a broad range of pH (Miller and Alper, 2019). Since the genome of *Y. lipolytica* was unraveled long ago and the tools for manipulating genomes and the knowledge of genetic engineering has also progressed dramatically, *Y. lipolytica* has become a suitable representative model organism for the production of natural biosynthetic products in the laboratory (Ledesma-Amaro and Nicaud, 2016). In this context, the present study was aimed to evaluate the ability of *Y. lipolytica* in the production of FA-OHs of different lengths, for which two bumblebee FARs (Tupec et al., 2019) were expressed in this yeast. The *Blap*FAR4 from *B. lapidarius* is capable of preferentially catalyzing the production of shorter FA-OHs (14–16 carbons), while *Bluc*FAR1 from *B. lucorum* prefers acyl chains containing 18–26 carbon atoms.

In the present study, we engineered multiple strains of *Y. lipolytica* for a redesigned lipid metabolism, with genes encoding *Bluc*FAR1 and *Blap*FAR4, to produce the FARs and obtain the corresponding fatty alcohols. The expression of *Bluc*FAR1 (JMY7086) stimulated the production of 18–24 carbon atom-long fatty alcohols, presenting the highest fatty alcohol production among all the strains (166.6 mg/L). Unlike JMY7086, the *Blap*FAR4-expressing strain (JMY7090) produced only 14.6 mg/L of fatty alcohols with a chain length of 16 carbons.

## Materials and Methods

### Strains, Media Composition, and Culture Conditions

All the strains of *Escherichia coli* and *Y. lipolytica* used in the present study are listed in Table 1. The recombinant strains of *Y. lipolytica* were constructed by engineering the wild type strain W29 (ATCC 20 460). The *E. coli* strains were cultured in a lysogeny broth medium containing a suitable antibiotic (100 μg/mL of ampicillin or 50 μg/mL of kanamycin), according to the standard protocol described by Sambrook and Russell (2001). Minimal YNB, YNBUra, and YNBLeu media agar plates were used for the selection of *Y. lipolytica* transformants. The minimal YNB medium comprised 1.7 g/L yeast nitrogen base (without amino acids and ammonium sulfate; BD, Erembodegem, Belgium), 5 g/L NH4Cl, 50 mM phosphate buffer (pH 6.8), and 20 g/L glucose. The YNBUra and YNBLeu media were prepared by adding 0.1 g/L of uracil and leucine, respectively, to the YNB medium. The agar plates were prepared by adding 20 g/L agar to the respective medium. A rich YPD medium containing 10 g/L yeast extract (BD, Erembodegem, Belgium), 10 g/L peptone (BD, Erembodegem, Belgium), and 20 g/L glucose (Mikrochem, Pezinok, Slovakia) was prepared for obtaining the inoculum of *Y. lipolytica*. The medium for lipid production (MedA^+^) comprised 1.5 g/L yeast extract, 0.5 g/L NH_4_Cl, 7 g/L KH_2_PO_4_, 5 g/L Na_2_HPO_4_.12H_2_O, 0.1 g/L CaCl_2_, 1.5 g/L MgSO_4_.7H_2_O, 10 mg/L ZnSO_4_.7H_2_O, 0.6 mg/L FeCl_3_.6H_2_O, 0.07 mg/L MnSO_4_.H_2_O, and 0.04 mg/L CuSO_4_.5H_2_O. The carbon source used was either glucose or crude glycerol (Mikrochem, Pezinok, Slovakia) in the concentration of 60 g/L. Owing to its high C/N ratio, this medium was suitable for the accumulation of lipids in yeasts. The MedA^+^ growth medium was prepared by modifying the MedA medium reported by Holdsworth et al. (1988). The yeast inoculum was prepared in 20 mL of the YPD medium in 100 mL flasks. Subsequently, 50 mL production medium in 250 mL baffled flasks was inoculated with a 24-h inoculum which had an optical density (OD_600_) of 0.1. The cells were cultured at 28°C and 130 rpm inside an orbital shaker (Innova 40R, NB, Canada). In order to confirm fatty alcohol production, the strains of *Y. lipolytica* were cultured for 3 days, while the cultivation of the selected strain continued for 5 days, with 24-h interval sampling, to describe the kinetics of fatty alcohol formation. All the experiments were performed in three independent biological replicates.

**TABLE 1:** The *Escherichia coli* and *Yarrowia lipolytica* strains and plasmids used in the present study.

**Strain (host strain)**	**Plasmid/genotype**	**References**
***Escherichia coli***		
JME1046	*JMP62-pTEF-URA3ex*	Lazar et al. (2013)
JME2563	*JMP62-pTEF-LEU2ex*	Dulermo et al. (2017)
JME2607	*JMP62–8UAS-pTEF-RedStar2-LEU2ex*	Dulermo et al. (2017)
JME3048	*JMP62–8UAS-pTEF-URA3ex*	Dulermo et al. (2017)
JME4149	*JMP1046-BlucFAR1*	This work
JME4151	*JMP1046-BlapFAR4*	This work
JME4303	*JMP3048-BlucFAR1*	This work
JME4305	*JMP2607-BlapFAR4*	This work
***Yarrowia lipolytica***		
W29	*MATA, wild type*	Barth and Gaillardin (1996)
Po1d	*MATA leu2–270 ura3–302 xpr2–322+pXPR2-SUC2*	Barth and Gaillardin (1996)
JMY6697	*Po1d, pTEF-BlucFAR1-URA3ex, LEU2*	This work
JMY6698	*Po1d, pTEF-BlapFAR4-URA3ex, LEU2*	This work
JMY3501	*W29 ura3–302 leu2–270 xpr2–322 Δpox1–6 Δtgl4+pXPR2-SUC2+pTEF-DGA2-LEU2ex+pTEF-GPD1-URA3ex*	Lazar et al. (2014)
JMY3820	*W29 ura3–302 leu2–270 xpr2–322 Δpox1–6 Δtgl4+pXPR2-SUC2+pTEF-DGA2+pTEF-GPD1*	Lazar et al. (2014)
JMY7086	*JMY3820, 8UAS-pTEF-BlucFAR1-URA3ex, LEU2*	This work
JMY7090	*JMY3820, 8UAS-pTEF-BlapFAR4-LEU2ex, URA3*	This work
JMY7094	*JMY3820, 8UAS-pTEF-BlucFAR1-URA3ex, 8UAS-pTEF-BlapFAR4-LEU2ex*	This work

### Plasmid and Strain Construction

The genes *BlucFar1* and *BlapFar4* were codon-optimized for *Yarrowia lipolytica* (Supplementary Figure 1). The synthetic fragments were digested using BamHI/AvrII, followed by insertion into the corresponding BamHI/AvrII sites of the already-available plasmids JME2607 and JME3048, which contained the *8UAS-pTEF* promoter (Dulermo et al., 2017). The *JMP62-pTEF-LEU2ex* and *JMP62-pTEF-URA3ex* vectors were employed to complement the LEU and URA auxotrophy, respectively, in the final strain. The plasmids were digested with NotI prior to being used for *Y. lipolytica* transformation using the lithium acetate method (Le Dall et al., 1994). The transformants were selected on the YNBUra, YNBLeu, or YNB media, depending on their genotypes. Subsequently, the genomic DNA was derived from the yeast transformants as described by Querol et al. (1992). The positive transformants were confirmed using PCR. The PCR amplifications were performed in an Eppendorf 2720 thermal cycler using GoTaq DNA polymerases (Promega). The obtained PCR fragments were purified using a QIAgen Purification Kit (Qiagen, Hilden, Germany), followed by verification through gel electrophoresis and sequencing. All the reactions were performed in accordance with the respective manufacturers’ instructions.

### Analytical Methods

In order to isolate the biomass, the cell suspensions were centrifuged (2,880 × *g*, 5 min), washed twice with the saline solution (NaCl, 9 g/L), and then once with deionized water, and finally freeze-dried. The freeze-dried cells were subjected to lipid analysis and dry cell weight (DCW) determination. DCW was determined gravimetrically.

The residual glycerol amounts during growth profile analysis were determined by performing HPLC (Agilent Technologies, Santa Clara, CA, United States) using an Aminex HPX87H column (Bio-Rad, Hercules, CA, United States) coupled with an RI detector and a DAD detector. H_2_SO_4_ (5 mM) was used as the mobile phase with a flow rate of 0.6 mL/min, as described by Lazar et al. (2011).

The freeze-dried cells (approximately 10 mg) were added to a mixture of 1 mL CH_2_Cl_2_ (containing 0.1 mg of C13:0 as the internal standard) and 2 mL anhydrous methanolic HCl solution, and the suspension was incubated at 50°C for 3 h. After the incubation, 1 mL of water and 1 mL of hexane were added, and the whole suspension was vortexed vigorously. The organic layer containing fatty alcohols and fatty acid methyl esters (FAME) was separated through centrifugation (2,880 × *g*, 5 min) and analyzed using GC-6890 N (Agilent Technologies, Santa Clara, CA, United States). The samples (1 μL) were injected automatically into the DB-23 column (50% cyanopropyl-methylpolysiloxane, length 60 m, diameter 0.25 mm, film thickness 0.25 μm) and analyzed. The analysis conditions were: carrier gas–hydrogen, inlet (230°C; hydrogen flow: 37 mL/min; split–10:1), FID detector (250°C, hydrogen flow: 40 mL/min, air flow: 450 mL/min.), gradient (150°C–0 min; 150–170°C–5,0°C/min; 170–220°C–6,0°C/min; 220°C–6 min; 220–230°C–6°C/min; 230°C–1 min; 230–240°C–30°C/min; 240°C–6 min). The chromatograms were analyzed using the Agilent Open LAB CDS software. The fatty alcohols and fatty acids were quantified according to the individual peak area normalized with the internal standard (C13:0). Individual fatty acids were identified according to the C4–C24 FAME standard (Supelco, Bellefonte, PA, United States). The fatty alcohol standards were obtained from Nu-Chek Prep and Sigma-Aldrich. GC-MS (EI at 70 eV) was performed to confirm the identity of the obtained peaks according to their MS spectra.

## Results

### Insertion of FAR Genes Into Wild Type *Y. lipolytica*

The genes *BlucFAR1* from *B. lucorum* and *BlapFAR4* from *B. lapidarius* were overexpressed, under the control of the *pTEF* promoter, in the genetic background of Po1d strain (Supplementary Figure 2). Po1d was constructed from the wild type strain W29 in an earlier study (Barth and Gaillardin, 1996). Both new strains JMY6697 (*Bluc*FAR1) and JMY6698 (*Blap*FAR4) were cultured in YPD and two MedA^+^ media, supplemented with either glycerol or glucose as the carbon source. The W29 strain was used as a control as it has the same genetic background as the host Po1d. All the strains were quite similar in terms of growth and did not differ significantly regarding the amount of accumulated lipids. The lowest lipid accumulation was obtained with the YPD medium, which is consistent with the assumption that the YPD medium is not suitable for lipid overproduction as it does not have a high C/N ratio. In both the MedA^+^ media, the lipid accumulation amounts were similar (Supplementary Table 1). However, trace amounts of FA-OHs were observed only under oleaginous conditions. The JMY6697 strain (*Bluc*FAR1) produced saturated FA-OHs with a chain length of 18 or more carbon atoms, while the JMY6698 strain (*Blap*FAR4) produced saturated FA-OH with a 16 carbon atom-long chain only. No FA-OHs were secreted into the medium. Since insect FA-OHs were produced in low quantities in these strains, it was decided to construct *Y. lipolytica* strains with further improved lipid metabolism to achieve higher FA-OH production by the expressed FARs.

### Metabolic Redesigning of *Y. lipolytica* for Effective Fatty Alcohol Production

**FIGURE 1:**
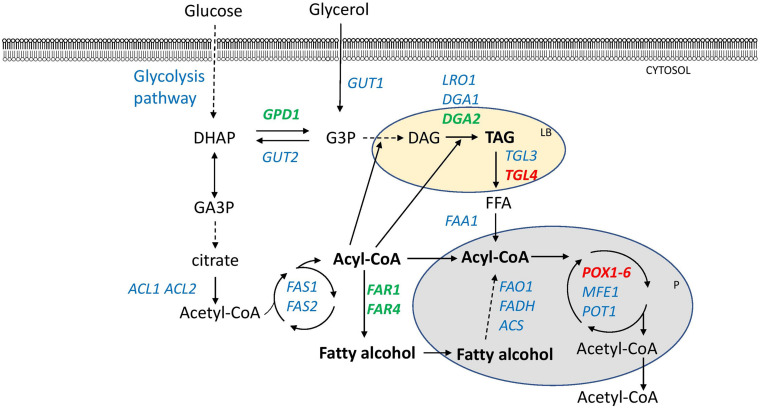
Lipid metabolism of manipulated *Y. lipolytica* strains producing fatty alcohols. Overexpressed genes are shown in green. Deleted genes are shown in red. Dashed lines indicate multiple steps. DHAP-dihydroxycetone phosphate, G3P-glycerol-3-phosphate, GA3P-glyceraldehyde-3-phosphate, DAG-diacylglycerol, TAG-triacylglycerol, FFA-free fatty acid, LB-lipid body, P-peroxisome. Enzymes: GPD1-glycerol-3-phosphate dehydrogenase, GUT1-glycerol kinase, GUT2-glycerol-3-phosphate dehydrogenase, ACL1-ATP-citrate lyase 1, ACL2-ATP citrate lyase 2, FAS1-fatty acid synthase 1, FAS2-fatty acid synthase, LRO1-phospholipid:diacylglycerol acyltransferase, DGA1 and DGA2-acyl-CoA:diacylglycerol acyltransferases 1, TGL3 and TGL4-intracellular lipases, FAA1-fatty acyl-CoA synthetase, FAO1-fatty alcohol oxidase, FADH-fatty alcohol oxidase, ACS-acyl-CoA synthase, POX1-6-acyl-CoA oxidases, MFE1-multifunctional enzyme, POT1-thiolase, and FAR1 and FAR4-fatty acyl-CoA reductases.

New strains capable of accumulating higher amounts of lipids were constructed. The JMY3820 strain, which had all the 6 *POX* genes and *TGL4* lipase deleted and the *DGA2* and *GPD1* overexpressed using the *pTEF* promoter, was selected as the host strain for FAR expression. The JMY3820 strain is an auxotrophic version of the JMY3501 strain. Both these strains were constructed from the JMY1233 (Beopoulos et al., 2008) strain in an earlier study (Lazar et al., 2014). In both these strains, the FARs were expressed under a stronger hybrid constitutive promoter named *8UAS-pTEF* (Dulermo et al., 2017). Therefore, in total, three new strains were constructed (Supplementary Figure 3): JMY7086 (*8UAS-pTEF-BlucFAR1*), JMY7090 (*8UAS-pTEF-BlapFAR4*), and JMY7094 (*8UAS-pTEF-BlucFAR1* and *8UAS-pTEF-BlapFAR4*). Changes in lipid metabolism are displayed in Figure 1.

### Biomass, Fatty Acid, and Fatty Alcohol Production

**FIGURE 2:**
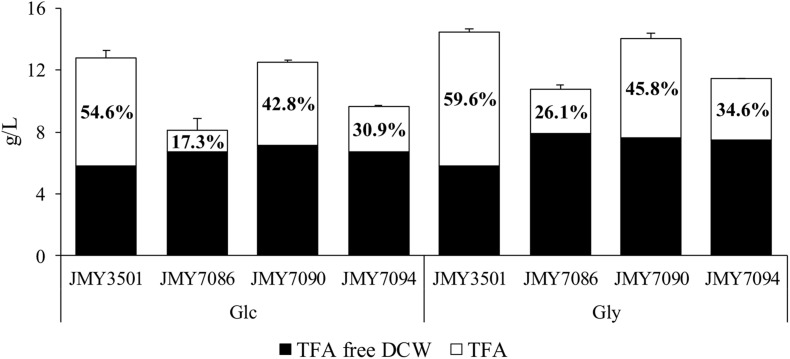
Biomass and lipid accumulation of JMY3501 (control), JMY7086 (*8UAS-pTEF-BlucFAR1*), JMY7090 (*8UAS-pTEF-BlapFAR4*), and JMY7094 (*8UAS-pTEF-BlucFAR1*, *8UAS-pTEF-BlapFAR4*) cultured in two different media. The yeast cells were cultured in a medium with a C/N ratio of 80 and 60 g/L of the carbon substrate. The carbon source was either glucose or glycerol. ■ Lipid free biomass (g/L) and □ total fatty acids, including fatty alcohols (g/L). The number inside the TFA is lipid accumulation, expressed as the TFA to DCW ratio. Each value is an average of the values obtained from three independent experiments.

The yeast strains were cultured in two MedA^+^ media with different carbon sources (glucose or glycerol) for 3 days. The C/N ratio of both the media was 80 and the concentration of carbon source was 60 g/L. The non-alcohol producing JMY3501 strain was cultured under the same conditions as the control for evaluating the influence of the alcohols on cell growth and lipid accumulation. Glycerol promoted higher biomass growth and total fatty acid including fatty alcohol accumulation (TFA) in all the strains, compared to glucose (Figure 2). Whether growing on glucose or glycerol, all the alcohol-producing strains produced quite similar amounts of lipid-free biomass, and the amount of lipid-free biomass produced by the control strain was lower compared to that of the alcohol-producing strains (5.8 g/L vs. approx. 6.8 g/L on glucose and 5.8 g/L vs. approx. 7.5 g/L on glycerol). The most remarkable difference was observed in the FAs accumulation in yeasts, which was inversely proportional to the FA-OH accumulation. The control strain JMY3501 accumulated approximately 60% of TFA/DCW, while the alcohol-producing strains accumulated only 17–45% of it. Even a higher production of fatty alcohols did not promote the secretion of fatty alcohols into the medium.

After 3 days of cultivation, all the yeast strains grown on glycerol accumulated more FA-OHs compared to the yeast growing on glucose (9.77 mg/g in JMY7086 on glycerol vs. 6.17 mg/g in JMY7086 on glucose) (Table 2). The titer of FA-OH was also higher on glycerol (104.78 mg/L on glycerol vs. 49.97 mg/L on glucose in JMY7086). The rates of the biosynthesis of individual FA-OHs facilitated by *Bluc*FAR1 reductase were different, although the final amounts of FA-OH produced were quite similar, as evidenced by the growth profile (Table 3). The JMY7086 strain (*Bluc*FAR1) could produce only saturated fatty alcohols with chain length ranging from C18 to C24. All the FA-OHs were produced in similar final amounts, with C22:0-OH being the most abundant one. When the *BlapFAR4* expression was driven by a stronger promoter *8UAS-pTEF* in the JMY7090 and JMY7094 strains accumulating higher amounts of lipids, an additional unsaturated FA-OH C16:1Δ^9^-OH was identified compared to the previous experiment in which only C16:0-OH was produced. The best FA-OH-producing strain was the JMY7086 strain expressing the *BlucFAR1* gene and yielding 9.77 mg/g DCW of FA-OH (104.78 mg/L). JMY7090 (*Blap*FAR4) produced the least amount of FA-OH at 1.04 mg/g DCW (14.6 mg/L). The expected higher FA-OH production in the JMY7094 strain expressing both the reductases could not be achieved as this strain produced only 5.68 mg/g DCW and 65.04 mg/L yield. On the basis of these results, only glycerol was selected for use as the carbon source, while JMY7086 was selected as the FA-OH-producing strain for subsequent experiments.

**TABLE 2:** Fatty alcohol production in JMY7086 (*8UAS-pTEF-BlucFAR1*), JMY7090 (*8UAS-pTEF-BlapFAR4*), and JMY7094 (*8UAS-pTEF-BlucFAR1*, *8UAS-pTEF-BlapFAR4*) cultivated for 72 h using different carbon sources.

	**JMY7086**	**JMY7090**	**JMY7094**	**JMY7086**	**JMY7090**	**JMY7094**
		
	**Glucose**			**Glycerol**		
**FA-OH (mg/g DCW)**						
C16:0-OH	–	0.13	0.53	–	0.68	1.65
C16:1Δ^9^-OH	–	0.09	0.18	–	0.36	0.49
C18:0-OH	0.82	–	0.44	2.58	–	0.93
C20:0-OH	0.82	–	0.66	2.09	–	0.78
C22:0-OH	2.02	–	1.56	2.93	–	1.13
C24:0-OH	2.51	–	0.93	2.17	–	0.70
Total	6.17	0.22	4.29	9.77	1.04	5.68
**FA-OH (mg/L)**						
C16:0-OH	–	1.66	5.09	–	9.61	18.92
C16:1Δ^9^-OH	–	1.07	1.74	–	4.99	5.64
C18:0-OH	6.66	–	4.27	27.69	–	10.62
C20:0-OH	6.61	–	6.35	22.38	–	8.96
C22:0-OH	16.33	–	15.05	31.44	–	12.94
C24:0-OH	20.36	–	9.02	23.26	–	7.96
Total	49.97	2.73	41.52	104.78	14.60	65.04

**TABLE 3:** Kinetics of the individual fatty acids and fatty alcohols in *Y. lipolytica*.

**Strain**	**JMY7086**					**JMY3501**				
**Time (days)**	**1**	**2**	**3**	**4**	**5**	**1**	**2**	**3**	**4**	**5**
	
**FA**	**FA (mg/g DCW)**									
C14:0	0.33	0.32	0.42	0.44	0.45	0.36	0.83	1.10	1.30	1.35
C15:0	0.20	0.18	0.54	0.69	0.83	0.22	0.37	0.80	0.95	1.09
C16:0	26.48	25.90	42.65	45.51	48.83	32.49	81.38	123.88	136.32	139.45
C16:1Δ^9^	5.64	4.09	5.19	5.29	5.30	7.46	23.92	40.11	44.46	44.74
C16:0-OH	0.00	0.00	0.00	0.00	0.00	0.00	0.00	0.00	0.00	0.00
C17:0	0.51	0.54	0.62	0.76	0.85	0.67	1.03	3.26	3.65	3.77
C16:1 Δ^9^-OH	0.00	0.00	0.00	0.00	0.00	0.00	0.00	0.00	0.00	0.00
C18:0	28.06	50.83	69.34	80.88	81.26	30.13	48.59	53.24	58.55	56.07
C18:1Δ^9^	57.62	66.54	93.81	97.81	96.68	65.84	180.29	323.85	347.97	341.47
C18:1Δ^11^	0.63	0.68	0.80	0.89	0.94	0.75	1.63	2.70	2.96	3.08
C18:2Δ^9,12^	19.18	19.31	24.04	26.01	30.72	18.01	18.14	27.88	29.69	31.93
C18:0-OH	0.00	0.54	2.12	4.72	1.59	0.00	0.00	0.00	0.00	0.00
C20:0	1.89	4.29	5.67	7.07	6.81	1.79	3.15	5.10	5.35	5.05
C20:0-OH	0.00	0.66	1.92	3.27	1.06	0.00	0.00	0.00	0.00	0.00
C22:0	2.11	5.25	4.60	5.95	5.04	1.83	2.91	3.81	4.13	3.86
C22:0-OH	0.00	1.57	3.05	4.41	2.71	0.00	0.00	0.00	0.00	0.00
C24:0	5.89	13.00	8.55	9.92	8.52	6.10	10.25	10.46	11.65	10.99
C24:0-OH	0.00	2.17	2.31	3.17	0.79	0.00	0.00	0.00	0.00	0.00
TFA	148.55	195.87	265.63	296.78	292.38	165.66	372.50	596.21	646.98	642.86

### Fatty Acid Profile

**FIGURE 3:**
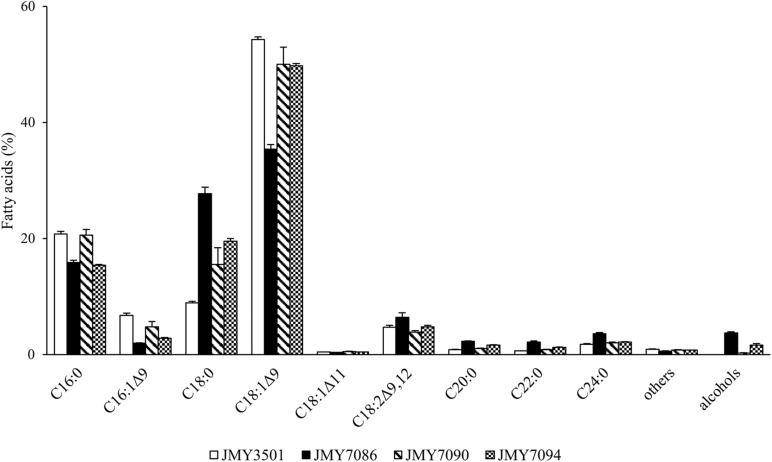
Fatty acid profiles of the strains JMY3501 (control), JMY7086 (*8UAS-pTEF-BlucFAR1*), JMY7090 (*8UAS-pTEF-BlapFAR4*), and JMY7094 (*8UAS-pTEF-BlucFAR1*, *8UAS-pTEF-BlapFAR4*) cultivated on glycerol. The column “others” includes fatty acids C14:0, C15:0, and C17:0. The column “alcohols” presents the total amount of all the alcohols produced together. The values provided are an average of the values obtained in three parallel experiments.

The FA profiles of the new strains and a control strain were compared (Figure 3). In all strains, oleic acid was determined as the major FA in the intracellular lipids. The FA analysis clearly demonstrated that the expression of FARs influenced the FA profile. The FAR-expressing yeast strains that produced higher amounts of FA-OHs presented a greater change in the FA profiles. The biggest change was observed for the JMY7086 strain, which accumulated the highest levels of FA-OH among all the strains. With the increasing FA-OH amount, the stearic acid content increased dramatically (8.9% in JMY3501 vs. 27.7% in JMY7086), while the oleic acid content decreased (54.3% in JMY 3501 vs. 35.4% in JMY7086). In addition, the palmitoleic acid level decreased and reached up to 1.9% in JMY7086 vs. 6.7% in JMY3501. When comparing JMY3501 and JMY7086, the ratio of FAs with chains longer than 20 carbon atoms rose by 2.6-fold for C20:0, 3.4-fold for C22:0, and 2-fold for C24:0. All these changes could be observed in all the FA-OH-producing strains, depending on the amount of FA-OH. The decrease in the palmitic acid level and increase in the linoleic acid level were obvious in JMY7086 and JMY 7094 (both expressing *Bluc*FAR1), while no such dependence was observed in JMY7090 (expressing only *Blap*FAR4).

### Daily Production Levels of the Individual Fatty Acids and Fatty Alcohols in the Strain JMY7086

**FIGURE 4:**
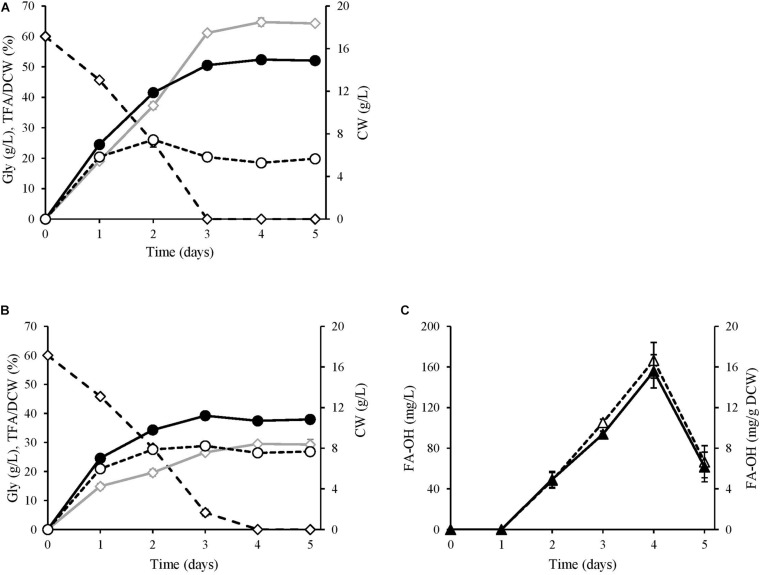
Kinetics of alcohol production. **(A)** JMY3501 (control) and **(B,C)** JMY7086 (*8UAS-pTEF-BlucFAR1*). Both the strains were cultivated for 5 days in a glycerol medium with an initial glycerol concentration of 60 g/L. Gly, kinetics of substrate consumption (◆); CW (cell weight), DCW biomass growth (•), TFA free DCW (∘), TFA, total fatty acid accumulation, including that of fatty alcohols (◆), FA-OH, fatty alcohols, mg/g DCW (▲), mg/L (Δ).

The alcohol-producing JMY7086 strain and the control strain JMY3501 were cultured in the MedA^+^ medium containing glycerol as a carbon source for 5 days, with sample retrieval every 24 h (Figure 4). After the first 24 h, no alcohol was detected in JMY7086, and the cells behaved similar to those in the control. Both the strains produced 7 g/L of DCW, of which a slightly lower FA content was accumulated in JMY7086 (15% in JMY7086 vs. 19% in JMY3501). The residual glycerol in the medium was 46 g/L. After 48 h, alcohol production had begun and differences among the strains could be observed. JMY7086 consumed less glycerol (22 g/L) compared to JMY3501 (25 g/L), the lipid-free biomass was approximately the same as that in the control (JMY3501 7.4 g/L vs. JMY7086 7.8 g/L), the DCW value was lower (JMY3501 12 g/L vs. JMY7086 10 g/L), and the accumulated TFA was less than that of the control strain (37% in JMY3501 vs. 20% in JMY7086). After 72 h, both the strains reached the respective stationary phases, and the amount of DCW reached its highest value and remained nearly the same for the next few days. Interestingly, the peak for the lipid-free biomass was observed on the second day in JMY3501, which decreased on the third day and then remained constant in the following days (approx. 5.6 g/L). Meanwhile, the amount of lipid-free biomass in JMY7086 increased to 8.2 g/L on the third day. At this time, glycerol was completely consumed by JMY3501, while 6 g/L of residual glycerol remained in the medium with JMY7086. The TFA accumulation reached its maximum value 24 h later, i.e., on the fourth day, with the FA-OH-producing strain accumulating less than half of the TFA amount accumulated by the control strain (65 vs. 29%). After the fourth day, no significant change in the lipid content was observed. The peak of FA-OH production was achieved on the 4th day. The yeast cells accumulated 5% FA-OH of TFA, which represented 15.6 mg/g DCW and a 166.6 mg/L titer of alcohols. On the 5th day, the FA-OH content decreased, which occurred most probably due to the depletion of the carbon substrate in the medium. In order to survive, the cells attempted to derive energy from the fat stored inside the lipid bodies. The deletion of the genes encoding lipase (TFL4) and acyl-CoA oxidases (POX1–6) prevented the cells from consuming lipids, although the presence of functional fatty alcohol oxidase (FAO1) and fatty alcohol dehydrogenase (HFD4) continued converting the fatty alcohols into fatty acids.

The effect of fatty alcohols on fatty acid accumulation is best described in Table 3. On the first day of cultivation, no huge difference was observed between the two strains. While JMY7086 accumulated slightly fewer FAs, its FA profile was quite similar to the FA profile of JMY3501. On the second day and beyond, the differences between the strains began deepening. JMY3501 began accumulating higher amounts of palmitic acid, palmitoleic acid, and oleic acid, while the increased amounts of palmitic and oleic acids accumulated in JMY7086 were much lower than those in the control. In addition, from the second day onward, JMY7086 did not accumulate palmitoleic acid at all. On the contrary, JMY7086 exhibited an increased amount of accumulated stearic acid, which was higher than that of the control. Although the relative contents of other FAs were different, the absolute amounts did not differ significantly.

## Discussion

Fatty alcohol production using microorganisms could reduce the production costs for insect pheromones and render the production process environmentally safer (Borodina et al., 2018b; Petkevicius et al., 2020). Although the literature has reported several approaches for producing fatty alcohols using *E. coli* and *S. cerevisiae*, one promising tool is the use of oleaginous microorganisms. So far, among all the oleaginous yeasts, *Y. lipolytica* is the most researched one (Borodina et al., 2018a). Host systems, either bacterial (*E. coli* or cyanobacteria) or yeast (*S. cerevisiae*, *R. toruloides*, or *Y. lipolytica*) that express non-insect enzyme sequences are reported to have reached several grams of fatty alcohols per liter of cultivation medium after optimization (Tupec et al., 2017). A fatty alcohol titer of 6.33 g/L was achieved in fed-batch fermentation in *E. coli via* the deletion of all fatty acyl-CoA thioestarases and *ldhA*, *pta*, and *ackA* genes, to starve cells of fatty acids and remove competing pathways (Liu et al., 2016). The expression of *Tyto alba* FAR (TaFAR1) in *Y. lipolytica* resulted in the production of 690 mg/L of hexadecan-1-ol (Wang G. et al., 2016), while the *Y. lipolytica* transformed with the *MhFAR* gene encoding the reductase from *Marinobacter hydrocarbonoclasticus* produced 6 g/L of alcohols representing an accumulation of 36 mg alcohols/g of cells (Cordova et al., 2019). The same reductase stimulated the production of 770 mg/L of fatty alcohols in *L. starkeyi* (Wang W. et al., 2016) and 8 g/L of fatty alcohols in *Rhodosporidium toruloides* (Fillet et al., 2015). Since the pheromone blends often contain unusual components, it is an advantage to use insect fatty acyl biosynthetic enzymes for their production.

In the present work, *Y. lipolytica* strains were engineered to have different genetic backgrounds with genes encoding reductases from *Bombus lucorum* (*BlucFAR1*) and *Bombus lapidarius* (*BlapFAR4*). When these genes were introduced into the Po1d strain under the control of the *pTEF* promoter, alcohols were detected only in trace amounts (in strains JMY6697 and JMY6698). Therefore, the second strategy was adopted, which involved redesigning the strain JMY3820, which had all the six *POX* genes and *TGL4* lipase deleted and the *DGA2* and *GPD1* overexpressed using the *pTEF* promoter, with the incorporation of *FAR* genes driven by a stronger promoter *8UAS-pTEF*. This genotype is particularly effective in accumulating high amounts of lipids (Dulermo et al., 2015). Deletions of *POX* genes and *TGL4* gene prevents degradation of storage lipids and oxidation of fatty acids. On the other hand, overexpression of the *DGA2* and *GPD1* drives the formation of the TAGs and incorporation of fatty acids into the TAGs. The premise was to implement the push and pull strategy (Tai and Stephanopoulos, 2013), with the higher lipid production as the driving force for the higher fatty acid synthesis. With the higher fatty acyl-CoA pool, more fatty acyl-CoAs can be redirected to the reduction by FARs. As the cells become more saturated with TAGs, more fatty acyl-CoA becomes available as a substrate for reductases. This strategy produced three new strains: JMY7086 (*8UAS-pTEF-BlucFAR1*), JMY7090 (*8UAS-pTEF-BlapFAR4*), and JMY7094 (*8UAS-pTEF-BlucFAR1*, *8UAS-pTEF-BlapFAR4*). A co-expression of both the reductases (JMY7094) did not meet the expectations. It might have affected the FA profiles or reduced the activity of the elongases (Figure 3). This could have led to limited availability of the substrate and ultimately a lower yield of alcohols. For instance, co-expression of *Bluc*FAR1 and *Blap*FAR4 resulted in a higher level of C16:0-OH and C16:1Δ^9^-OH and a decreased level of longer FA-OHs (Table 2). Another explanation could be as a consequence of an increased degree of FA-OH degradation. Table 3 presents the degradation of long-chain fatty alcohol between Day 4 and Day 5, which might have been induced by the presence of C16 fatty alcohols, which would also explain the lower level of total FA-OHs in JMY7090. It may also be interpreted by the level of *Bluc*FAR1 expression, possibly due to the integration site of *Bluc*FAR1 in the double mutant or titration of the *pTEF* promoter. All of the results did not indicate alcohol toxicity on the cells and rather indicated the inhibition of FA formation and lipid accumulation by the alcohols or the by-products of their metabolism. It is also possible that in addition to being affected by the total FA-OH amount, the FA profile is affected by the FA-OH composition as well. For example, Figure 3 clearly shows that the JMY7086 strain produces more long chain fatty acids (>C18). Indirectly, this tells us that the production of long-chain fatty alcohols may well be driving the elongation of fatty acids - the so-called metabolic driving force. This also corresponds to the push and pull strategy described by Tai and Stephanopoulos (2013). If FAR is taking substrates (long-chain fatty acyl-CoAs) from lipid metabolism, the cells are overcompensating by producing more long-chain fatty acyl-CoAs. Although the metabolic engineering of the selected *Y. lipolytica* strains enabled the production of the desired FA-OHs, there is scope for further improvement. A combination of lipid metabolism manipulation in *Y. lipolytica* and engineering of the FA-OH metabolism could produce higher amounts of specific FA-OHs synthesized by a particular FAR in low concentrations. It is well recognized that in certain cases, *Y. lipolytica* secretes fatty acids and fatty alcohols into the medium, which increases the production while simplifying the purification. Most often, this occurs spontaneously when the cells accumulate high amounts of toxic products that they are unable to degrade. The excretion of fatty alcohols may also be achieved by optimizing the fermentation process. The deletion of *FAO1* (fatty alcohol oxidase) and *HFD4* (fatty alcohol dehydrogenase) could prevent product degradation by the cells (Dahlin et al., 2019; Holkenbrink et al., 2020). Another approach is to manipulate the *FAA1* gene (fatty acyl-CoA synthetase) or the lipases to increase the substrate availability for the functioning of FARs, or to use dodecane-mediated extraction fermentation for the secretion of alcohols into the medium (Cordova et al., 2019).

In a previous study, the FARs used in the present work were expressed in *Saccharomyces cerevisiae* (Tupec et al., 2019), and several discrepancies were observed between the composition of FA-OH synthesized by the FARs in labial gland of bumblebee and in the *S. cerevisiae* expression system. The reductase *Blap*FAR4 produced poly-unsaturated fatty alcohols in the yeast cells, which are not present in the labial gland of male *B. lapidarius*. It is possible that a pool of fatty acyl CoA precursors in the yeasts differs from those present in the labial gland. The concentrations of C18:1Δ^9^-CoA, C18:2Δ^9,12^-CoA, and C18:3Δ^9,12,15^-CoA in the labial gland of *B. lapidarius* might be extremely low, to the extent that detectable accumulation of the corresponding alcohols is not possible. This implies that the ratio of fatty alcohols in the pheromones is dependent not only on the FAR substrate specificity, rather also on substrate availability, which is affected greatly by the other mechanisms such as the conversion of fatty acyls into fatty acyl-CoAs, fatty acyl-CoAs hydrolysis, etc. The differences in the quantity and ratio of fatty alcohols between *Y. lipolytica* and *S. cerevisiae* (Table 4) most likely reflects the differences in metabolism of these species. While *Y. lipolytica* is considered as oleaginous yeast, *S. cerevisiae* do not accumulate significant quantities of lipids. FA composition of lipids of these two yeast species is also different. While *Y. lipolytica* naturally prefers to accumulate the FAs with a chain length of 18 carbon atoms, *S. cerevisiae* prefers 16 carbon atom-long FAs. It could, therefore, be assumed that this difference in the accumulation of FAs was reflected in the fatty acyl-CoA pool as well. Since the fatty acyl-CoA pool is the source of substrates for FAR, a higher quantity of either 16 or 18 carbon long FA could influence the quantity and quality of FA-OHs. While the enzyme *Bluc*FAR1 produced the highest FA-OH content in *Y. lipolytic*a (166.6 mg/L), the same enzyme produced only a small amount of FA-OH in *S. cerevisiae* (6.9 mg/L). On the contrary, *Blap*FAR4 worked much better in *S. cerevisiae* (79 mg/L) than in *Y. lipolytica* (14.6 mg/L). Therefore, variations in yields could be attributed to the difference in the substrate availability between *Y. lipolytica* and *S. cerevisiae*. However, the expression level of the enzymes and in particular their activities also might have an effect on the synthesis of FA-OH.

**TABLE 4:** Comparison of pheromone production between *Y. lipolytica* and *S. cerevisiae* expressing the same genes.

	***Bluc*FAR1**		***Blap*FAR4**	
	**JMY7086**	***S. cerevisiae***	**JMY7090**	***S. cerevisiae***
	
	**Fatty alcohols (mg/L)**			
C16:0-OH	–	0.1	9.61	35
C16:1-OH	–	–	4.99	44
C18:0-OH	50.5	3.3	–	–
C20:0-OH	35.0	1	–	–
C22:0-OH	47.2	1.3	–	–
C24:0-OH	33.9	0.7	–	–
C26:0-OH	–	0.5	–	–

Taken together, the results of the present work demonstrated that bioengineered *Y. lipolytica* strain with deleted *POX* and *TGL4* lipase genes and overexpressed *DGA2* and *GPD1* genes is suitable for the efficient production of FA-OHs with different chain lengths. To our knowledge, this is the first study showing the ability of *Y. lipolytica* to produce FA-OHs of different lengths and the first study achieving production of FA-OHs longer than 20 carbons by *Y. lipolytica*.

## Data Availability Statement

The raw data supporting the conclusions of this article will be made available by the authors, without undue reservation.

## Author Contributions

IP, RL-A, J-MN, and MC defined the concept of the study. MT isolated the genes. IP provided the gene sequences. RL-A designed and prepared the constructs. PG and JH designed and performed the cultivation experiments and performed the GC MS analyses. JH prepared the manuscript. IP, MT, RL-A, J-MN, MC, and PG revised the manuscript. All authors contributed to the article and approved the submitted version.

## Conflict of Interest

The authors declare that the research was conducted in the absence of any commercial or financial relationships that could be construed as a potential conflict of interest.
