# Supplementary material for: Production of Long Chain Fatty Alcohols Found in Bumblebee Pheromones by Yarrowia lipolytica
Source: Front Bioeng Biotechnol. 2021 Jan 8;8:593419. doi: 10.3389/fbioe.2020.593419 (PMC7820814; doi:10.3389/fbioe.2020.593419)
Supplement: Supplementary file 1 [file Data_Sheet_1.PDF]

Supplementary Material

BlucFAR1

ccccGGATCCGGTCTCtAATGAACACTGAGTTCACTGAAAAGTCTAACAAGGTCAACTCCATCGAAGGTTTCTA
CGCTGGTACAGGTATCTTTATCACAGGTGCCTCAGGTTTTGTCGGTAAAGGTTTGTTGGAAAAGTTGATCAGA
GTTTGTCCTAGAATCGTTGTATTATTCATCTTGGTTAGACCAAAGAAACATCAAACAATGGAACAAAGATACA
AGGAAATCATGGATGACCCTATCTTTGATGACATCAAAGCTAAGAATCCATCCGCATTGAAAAAGGTCCATCC
TGTTGAAGGTGACATTTCCTTACCAAAGTTGGGTTTGAGTCAAGAAGATAGAAACATGTTGATAGAAAACGT
CAACATCTTGTTTCACGTTGCTGCATCTTTGAACTTCAAGGAACCATTGAACGCCGCTGTAAATACTAACGTCA
AGGGTACATTTTCTATAATCGAATTGTGTAACGAATTGAAGCATGTTATATCAGCTGTACACGTCTCTACAGC
ATATTCAAATGCCAACTTGCCTGAAATAGAAGAAAAGGTTTACTCCACTATCTTACAACCATCTTCAGTAATTG
AAACATGCGACAGTTTGGATAAGGAATTGATTAAGTTGTTGGAAGAAAGAATTTTGAAAATACATCCTAACA
CGTACACCTTCACGAAGAATTTGGCAGAACAAATCTTGTCCAGTTCTTCAACTAACTTCCCAATAGCAATCGTT
AGACCTTCTATCATTTCCGCCAGTTTAAAAGAACCATGTCCTGGTTGGTTGGGTAATATTACAGCCCACATAGC
TTTGGGTTTGTTTATTTCAAGAGGTTTCGCCAAGATCACCTTAGCTAACCCTGACACTATCACAGATACCGTAC
CATTAGACTATGTCGTTGATACAATTTTGTGTGCAGCCTGGCATGTTACCTTGCACAGAGATATGAACGTTAA
GGTATACAACTGCACCAATAACGCCAGACCAATTAATTACGGTGAATTGAAGGACACTTTTGTCAAGTACGCT
ATTCAAATACCTATGGATGGTTTAGTTTGGTATCCATGTTGCGCAATGGTTTCTAACAGATACGTATACTCAAT
CTTGACCTTATTCTTGCATACTTTGCCTGCTTTTATTATGGATATTTTCTTAAGATTGCAAGGTTCAAAGCCAAG
AATGATGAAGATCTCTAAGTACTACGATACAATGTCAATCGTTACCAACTACTTCTCCACTAGACAATGGAGT
TTCAAAAAGGATAACGTTATTAATATGATGAAAGAAGTCAAGACTTTGGAAGATTCTGACATCGTTAGATTAG
ATTTGCAAGATATGGACTGGGATAAGTACATCGCTATATGCGTTATCGGTATCAAAAAGTTTATTTTCAAAGA
AGACCCAAAGTCCTTAGATGCTGCATTGAGAAGATTGAGTATCTTTTACTGGATTCATCAAATGACTAAAGCC
TTCGCTATTATTATCTTATTGACCATTATTTTGAGAGTTATGTATTGATCTAaGAGACCCCTAGGcccc

BlapFAR4

ccccGGATCCGGTCTCtAATGGATACAATCAATAAAGAAAGGAATGAAAATACGATCAATAAAGGGTTGAATA
AGACGAATACTCTCGAAGAGTTCTACGCTGGTAGTGGGATTCTTGTGACTGGAGCAACCGGTTTCGTTGGAA
AAGGTCTCTTGGAAAAACTGATCCGCGTGTTTCCTCGCATTGCTGCCATTTTTATACTACTGCGTCCAAAAACG
GACGAAACGATAGAACAACGATTTAAGAAGCTCATAGATGATCCCTTTTACGATGCCATCAAAGCAAAGCAC
CCCACGGTTTTCAGCAAAGTTTATCCCGTGAAAGGTGACGTGAATCTGCCAGATTTAGGTCTTTCGCGAGAAG
ATAGAAATCTGCTGTTAGAGAATGTAAATATAGTGTTTCATGTCGCGGCCACTGTGAGATTCGACGAGCCGTT
ACACGTGGCAGTTAATGTGAATACTAATGGTACTGGTCGTGTTATCGAACTTTGGAACGATCTGAGACATCCG
ATTAGCTTCGTTCACGTTAGCACAGCTTATAGTAATGCGAATCTACGTGAGATCGAGGAAAAAGTTTATACTA
CGAGCTTGAAACCTGCTGATGTGATCGATATGTGTGACAAATTGGACAAAACTTCCATCAACGAAATAGAGA
AAACGATTTTAAAAACTTATCCAAATACATACACATTCAGTAAGAATTTAGCAGAGCAGATTGTAGCAAGCAA
GAGTAAACACCTGTCAGTTGCGATAGTACGACCAAGCATAATTGGTGCCTCGTTGGCAGAACCATGTCCCGG
TTGGATAGAGAATATTTCTGCATTTACAGATAGCTTCGTGCTAATCGGCAGAGGATGTGCAACAGCGATACG
AGGTAGGAGAGATGCGAGAATGGATATAGTGCCTGTCGACTTCGTAGTCGATACGATAATTTGTACCGCATG
GCATGTCACGTTACACAGTGATCATGAAGTTAAAGTTTACAACTGCACGAGCAACGCATACCCTTTTAAATGG
GGTCAGATGAGAGATGCCATTGTAAAATGTAGCATAGAATCGCCACTGAACAATACGCTATGGTACCCGGGT
TGTCCAATGATAGCTAATAGATATATTTACAACGTTCGGAGTGTAATTCCGCATGTTTTTCCTGCGTTCATCAT
AGATATATTTTTAAGACTTCGAGGTAGTAAACCAATAATGATGAAACTTCTCCAAAATGGCAATAAGCTGTTT
ACAGTGACAGCATATTTCGCTCTGAACGAATGGACTTTCCAAAAGGATAACTGTTTCGATTTAATGAGAAAAG

TGAAAATGTTGAACGACAGCGATATGGTCAGACTAGATTTACGGGATATGGATTGGGAGAAGTACGTTGCA
ATTTACCTGATGGGAATTAGGAAATTTATTCTGAAACAAGACTTTAATTCAACAGCCCAACAACGGTTATCAA
GGTTGTACTGGATAGATCAGATCACTAAAATATCCGGTATAATGATCTCACTACTGATGATATACTGTATCGTT
TATTGATCTAaGAGACCCCTAGGcccc

Supplementary Figure 1. Sequences of genes *BlucFAR1* and *BlapFAR4* coding bumble bee reductases optimized for expression in *Yarrowia lipolytica*. Gene sequences are marked yellow.

Supplementary Table 1. Comparison of biomass and lipid production among W29 (control), 6697 (BlucFAR1) and 6698 (BlapFAR4).

**Strain**	**YPD**		**Med A+ (glucose)**		**Med A+ (glycerol)**	
	DCW (g/L)	TFA (%)	DCW (g/L)	TFA (%)	DCW (g/L)	TFA (%)
W29	10,6±0,3	6,6±0,1	10,7±0,3	20,4±1,2	7,6±0,5	18,4±3,1
6697	11,6±0,3	6,8±0,1	10,9±0,1	22,1±0,5	7,8±0,05	22,6±10,1
6698	11,1±0,2	6,6±0,1	10,4±0,2	20,8±1,8	7,4±0,2	19,3±0,6

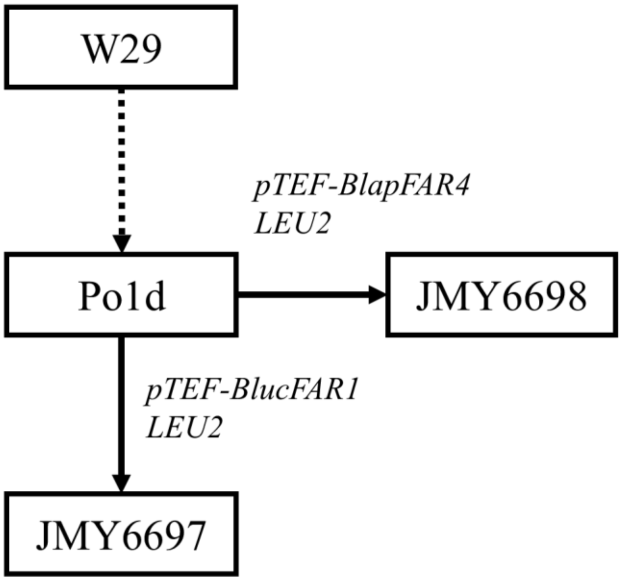

Supplementary Figure 2. Steps involved in the construction of fatty alcohol-synthesizing yeast cells. First, the auxotrophic strain Po1d (Leu$^-$ Ura$^-$) was constructed from the wild type strain W29. Po1d was further modified by inserting the *FAR* genes under the control of *pTEF* promoter, which provided two novel strains: JMY6697, which expressed the *BlucFAR1* gene, and JMY6698 which expressed the *BlapFAR4* gene. The dotted line indicates several steps, while the full line is for a single modification step.

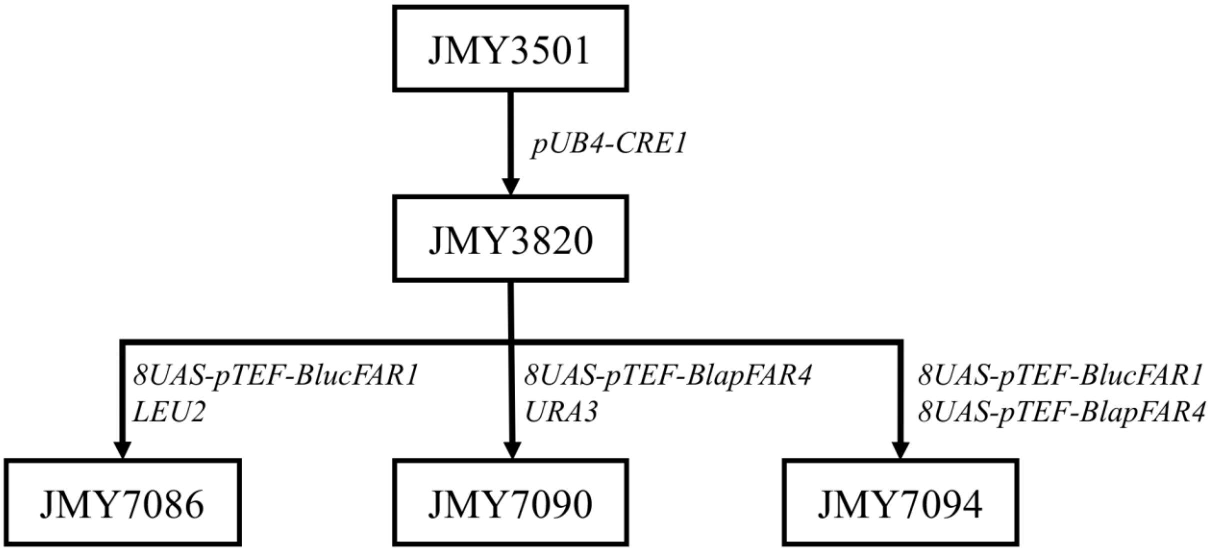

Supplementary Figure 3. Metabolic redesigning of yeast cells for effective fatty alcohol production. The construction began with the prototrophic strain 3501 (*Δpox1–6 Δtgl4 pTEF-DGA2-LEU2ex pTEF-GPD1-URA3ex*) which was derived from JMY1233 (Beopoulos et al. 2008). JMY3820 is identical to JMY3501, with the only difference being that the *URA3ex* and *LEU2ex* markers in the former were rescued as described in a previous report (Fickers et al., 2003). JMY7086 and JMY 7090 were obtained by introducing *BlucFAR1* and *BlapFAR4* genes, respectively, under the control of *8UAS-pTEF* promoter. JMY7094 was created by inserting both the FAR-coding genes simultaneously into JMY3820. The full line indicates a single modification step.

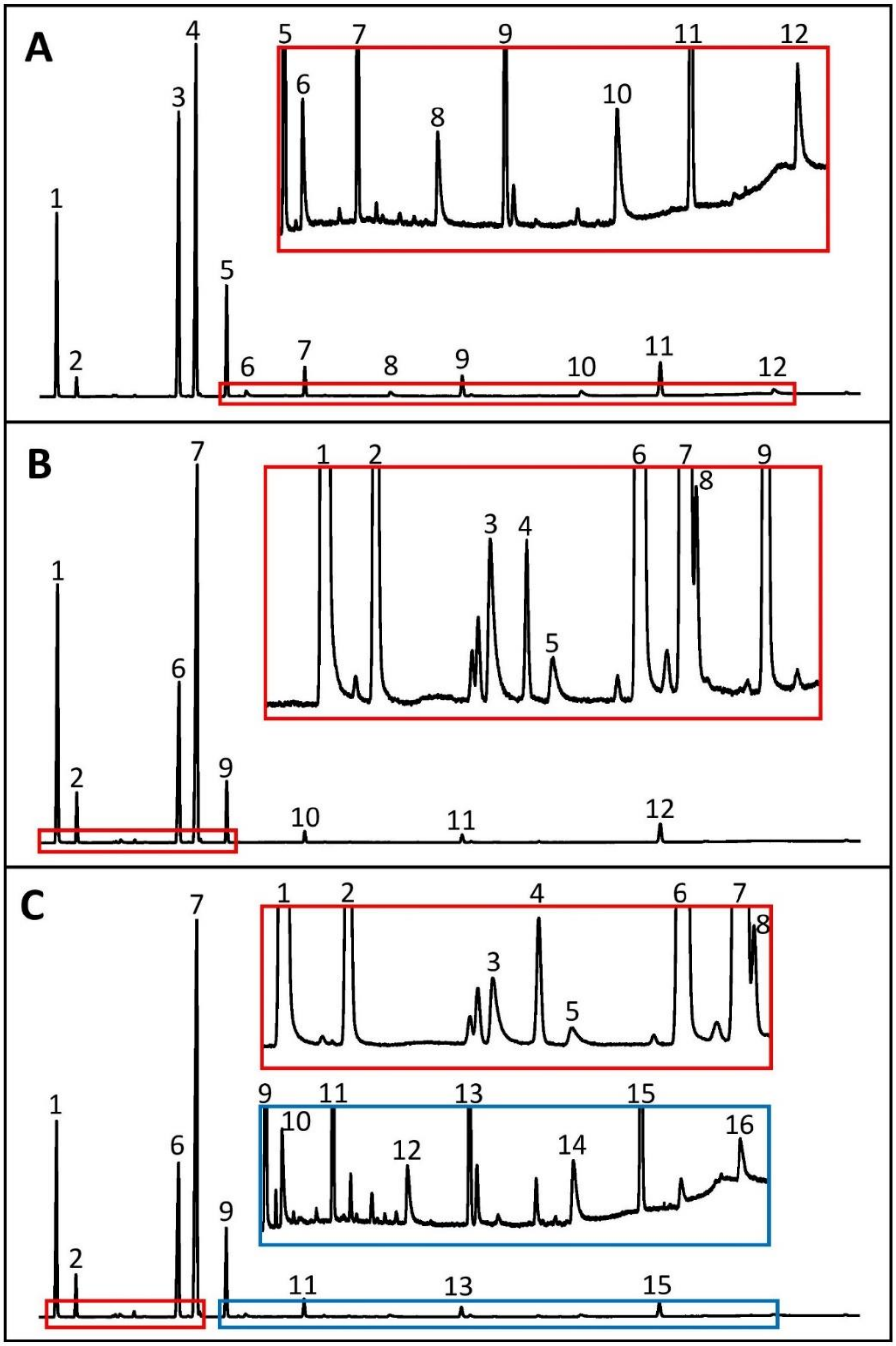

Supplementary Figure 4. Selected GC chromatograms of fatty acids and fatty alcohols analysis. The section of the chromatogram is displayed between 8.6 to 24 minutes. Red highlighted rectangles are zoomed areas displaying fatty alcohols.

A (JMY7086): 1 – C16:0, 2 - C16:1Δ^9, 3 – C18:0, 4 - C18:1Δ^9, 5 - C18:2$\Delta^{9,12}$, 6 – C18:0-OH, 7 – C20:0, 8 – C20:0-OH, 9 – C22:0, 10 – C22:0-OH, 11 – C24:0, 12 – C24:0-OH;

B (JMY7090): 1 – C16:0, 2 - C16:1Δ^9, 3 – C16:0-OH, 4 – C17:0, 5 - C16:1Δ^9-OH, 6 – C18:0, 7 – C18:1Δ^9, 8 – C18:1Δ^{11}, 9 – C18:2$\Delta^{9,12}$, 10 – C20:0, 11 – C22:0, 12 – C24:0;

C (JMY7094): 1 – C16:0, 2 - C16:1Δ^9, 3 – C16:0-OH, 4 – C17:0, 5 - C16:1Δ^9-OH, 6 – C18:0, 7 - C18:1Δ^9, 8 – C18:1Δ^{11}, 9 - C18:2$\Delta^{9,12}$, 10 – C18:0-OH, 11 – C20:0, 12 – C20:0-OH, 13 – C22:0, 14 – C22:0-OH, 15 – C24:0, 16 – C24:0-OH.
